# Exploring factors that influence the behavior response to novel object tests in young thoroughbred horses: investigating sex, test site and auction history

**DOI:** 10.3389/fvets.2024.1478350

**Published:** 2025-01-15

**Authors:** Lara Klitzing, Katharina Kirsch, Maria Schindler, Roswitha Merle, Gundula Hoffmann, Christa Thöne-Reineke, Mechthild Wiegard

**Affiliations:** ^1^Institute for Animal Welfare, Animal Behavior and Laboratory Animal Science, Freie Universität Berlin, Berlin, Germany; ^2^Institute for Veterinary Epidemiology and Biostatistics, Freie Universität Berlin, Berlin, Germany; ^3^Department Sensors and Modelling, Leibniz Institute for Agricultural Engineering and Bioeconomy (ATB), Potsdam, Germany

**Keywords:** animal welfare, behavioral test, equine behavior, horse, novel object test, thoroughbred racehorses

## Abstract

**Introduction:**

The novel object test is one of the three most common fear tests in veterinary science and employed in several different species. Although having been applied in several different studies in horses, it is surprising that there is no standardized test procedure available for these kinds of tests.

**Methods:**

This study investigated the performance of the novel object test on 42 young Thoroughbred horses to determine the effect of sex (mare or stallion), test sites (round pen or paddock) and whether the horses had previously participated in an auction or not on the behavior during the novel object test.

**Results:**

Differences in horses’ behavior during the novel object test were primarily attributed to the test sites. The animals showed significant (*p* < 0.05) intra-individual differences in the novel object test in the round pen and in the paddock. Sex did not affect the direct interaction with the novel object. The horses that had not participated in an auction seemed to actively perceive the novel object more quickly, so that the latency to first fixation on the object was significantly shorter.

**Discussion:**

In order to obtain comparable results, it is recommended that novel object tests should be performed at the same location and under identical conditions. Furthermore, it is important to consider the individual behavior of each horse.

## 1 Introduction

The novel object test is a behavioral test designed to investigate exploratory and fear behavior of unknown objects when animals are confronted with an unfamiliar, usually stationary object in a defined enclosure. The novel object test is typically conducted on individual animals (1). Novel object tests are used in many studies with horses for various purposes. In some studies, the novel object test is used to assess the character or temperament of horses (2–4) or, for example, to select a suitable horse for a rider (5). Under the premise that the way an animal reacts to a novel object can indicate its levels of stress and anxiety but also exploratory motivation, curiosity and play behavior, novel object tests can be used for the assessment of animal welfare (6, 7) or are part of pharmacological studies (8). However, the literature lacks a standardized procedure for performing novel object tests in horses. Novel object tests are typically conducted in indoor arenas like round pens (8, 9) or a training hall (10–12) or paddocks (13, 14). In some studies, horses are exposed to unfamiliar objects in their stables to prevent external factors, such as handling or social separation, from affecting the results (7, 15). However, the specific method used is not consistent. Moreover, the age and previous experience of the animal may influence behavior during the novel object test. It is, however, important to note that it is difficult to separate age from the experiences a horse has had throughout its life (14). According to Bulens et al. (7), younger horses exhibited more object-related behavior during a novel object test which might indicate that younger horses are more curious than older horses. Graf et al. (16) found that older horses showed less fearful behavior when confronted with different unknown stimuli than younger horses and Visser et al. (17) showed that the physiological stress response to a novel object test decreased with increasing age as indicated by heart rate which might be attributed to a higher habituation to different situations in older horses. Age and previous experience should therefore be taken into account when assessing behavior during novel object tests. In other studies, the age and sex of the horses had only a minor influence on the results of the novel object test, much more consideration was given to the genetics of the animals (7, 18, 19). It is well-established that horses are capable of discriminating between different colors and shapes of objects. However, they do not exhibit generalization between objects that differ in both color and shape (20). The housing system also effects behavior during the novel object test (7). Research has shown that box housing is associated with increased activity levels during the novel object test (18). A significant difference when implementing the novel object test can be noted when humans are present or not. Research has shown that horses tend to touch the novel object more quickly when they are led by a handler on a rope (13). In general, as a prey species, being vulnerable to various stimuli is essential for horses and different test conditions may therefore influence their behavior and thus the results of behavioral tests.

The novel object test is recommended by various institutions. For instance, the Wageningen Welfare Assessment Protocol for horses suggests a novel object test with one person restraining the horse on a rope while another person presents a Rubik’s cube (6). The German Veterinary Association has included the novel object test in the protocol for the mandatory examination of Thoroughbred racehorses before their first race (9). The purpose of this behavioral test is to evaluate the stress level of racehorses in addition to their general behavioral assessment in the husbandry system and during handling. The test is intended to evaluate the horses’ mental and physical capacity to cope with the demands of a race and associated preparations. To achieve this, a modified version of the fear test described in the AWIN Welfare Assessment protocol for horses was selected. During the fear test, the horse is presented with an unfamiliar object in its stall, such as a bottle filled with stones, which is suspended from the ceiling without movement and then dropped in the subsequent step (21). Testing horses in their home stable has the advantage that novel object tests can be easily performed and repeated (22) and that results are not influenced by changing the environment, social isolation or handling (7, 15, 23). However, the test described in the protocol for racehorses is conducted in an arena instead of a box, allowing the horse more freedom of movement to exhibit flight reactions (9).

The German Veterinary Association provides precise recommendations for the performance of the novel object test. According to these recommendations, the test should be performed in a familiar, enclosed area that is preferably covered. To ensure accurate results, it is recommended to avoid any visual or acoustic distractions from food, conspecifics, or people during the test. Additionally, it is emphasized that the test should be conducted in the same location before the horse’s first training and first race to ensure comparability of results (9). The veterinarian conducting the test evaluates the outcome of the test, taking into account the entire examination as well as the husbandry environment and decides whether the horse is physically and mentally fit to cope with the demands of training and racing.

Valid interpretation of novel object tests requires comprehensive knowledge of environmental and individual factors that might influence the behavior during a novel object test. To the best of the authors´ knowledge, previous studies have paid little attention to the influence of different environments, sex and the horses’ previous life experiences in terms of their behavior during the novel object test. The aim of this study therefore was to determine if the horse’s behavior during the novel object test and consequently the test results are impacted by the environment and whether there are sex-specific differences. Given that a horse’s previous experiences can impact its behavior, it is critical to consider this potential influencing factor as well.

## 2 Materials and methods

This study and its procedure were approved by the State Office for Consumer Protection of Saarland (Landesamt für Verbraucherschutz Saarland). It is not considered to be an animal experiment requiring authorization.

### 2.1 Animals

Forty-two Thoroughbred horses (19 stallions and 23 mares) were selected from a stable in Germany specialized in the preparation of young racehorses. The horses were housed in individual stalls, but were allowed to graze in small groups on pastures for at least 2 h on 5 days a week. They were fed on hay and stable’s own mixture of high-energy, racing-horse feed. The horses were between 23 and 27 months old and had experience in handling. The general handling of the horses included leading them by halter and rope or a bridle, as well as daily contact with the stable staff when feeding and cleaning the stalls. Additionally, the horses experienced regular veterinary and farrier care. The study was conducted in February. At the time of the study, 36 of the selected horses (18 mares and 18 stallions) had been in pre-training for approximately 4–5 months. The other 6 horses (1 stallion and 5 mares) had experience in handling, but had not yet been under saddle. Of the 36 horses that had already been under saddle, 24 had previously been at auction and 12 had not been at auction. Of the 6 horses that had not yet been ridden, 3 had been to an auction and 3 had not been to an auction. If horses are sold at auction as yearlings, they are usually accustomed to being bridled, transported and being housed in an unfamiliar environment. It is reasonable to assume that a horse that has been to an auction has gained significantly more experience, which may affect its behavior in a novel object test. On the other hand, it is also possible that the abrupt social separation and potentially unfamiliar handling practices during an auction lead to negative associations with novel environments which could potentially cause higher stress responses during a novel object test. Whether the horses have participated in an auction or not was therefore also taken into consideration. None of the horses had any experience in behavioral testing.

### 2.2 Implementation of novel object test

**Figure 1:**
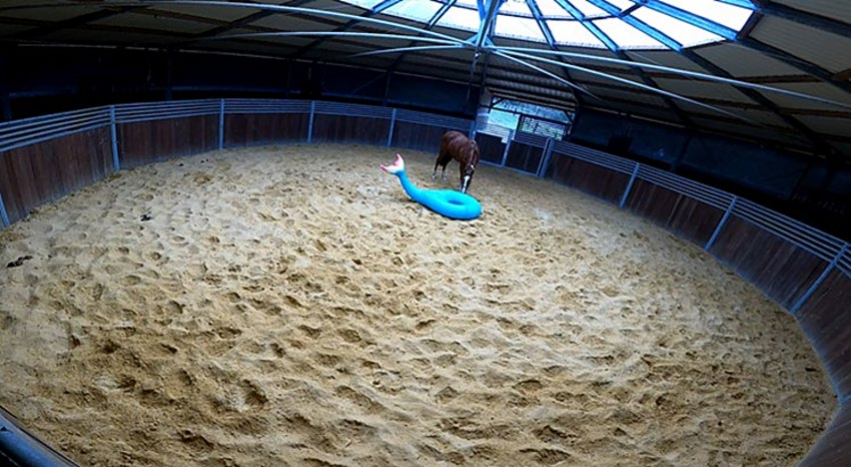
The novel object used during the test in the round pen; the pool raft shaped like a mermaid’s tail placed in the center of the testing site.

**Figure 2:**
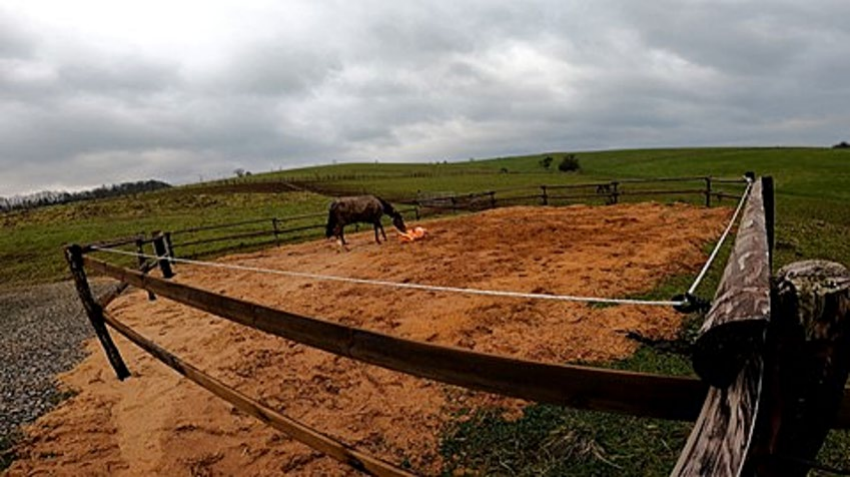
The novel object used during the test in a paddock; the pool raft ‘clown fish’ was placed in the center of the testing site.

Novel object tests were carried out in February 2023 at two different test sites. The first test site was a standard round pen (Figure 1) surrounded by a horse walker commonly found on horse farms. At a height of two meters, the round pen was surrounded by opaque wooden walls. The round pen had a diameter of 14 meters and was filled with a mixture of sand. The entire facility was covered with the roof in the center of the enclosure made of plexiglass, allowing for daylight to enter. The second test site for this study was a paddock (Figure 2). The sand paddock, with a side length of 18 by 18 meters, was surrounded by a wooden fence, including an electric wire, and a metal gate. The horses were familiar with the round pen and associated horse walker due to regular use during training. However, since both the stallions and mares were allowed to go out into large pastures in small groups, the horses were not fully accustomed to being kept alone in the round pen or on a single paddock.

All horses were tested once in the round pen. In addition, 12 horses (6 mares and 6 stallions) completed a second novel object test in the paddock 3 days later. Due to the limited amount of time that was available to conduct this study and to be able to test all horses on the same day in order to reduce the impact of different whether conditions on the results, only 12 horses were subjected to a second test in the paddock. In preparation for the novel object test, an inflatable pool raft was placed in the center of the test site. In order to rule out the possibility of familiarization with the object, two different pool rafts were used in the two different test locations. For the round pen, the pool raft in shape of a “mermaid’s tail” (approx. 170 × 85 × 100 cm) served as the unknown object, whereas the object “clown fish” (approx. 155 × 86.5 × 39 cm) was used in the paddock. The horses were led to the selected test site by a known person, turned with their heads toward the entrance, and then released from the rope and halter. The person left the test site immediately and a timer was set for 10 min once the entrance door was closed. The persons conducting the test moved out of sight so that the horses could not see or hear them to minimize their influence on the horses’ behavior during the novel object test. The horses were always in sight to enable the researchers to intervene quickly in case of an emergency. Two GoPro hero 11 black action cameras (GoPro, Inc., San Mateo, CA, USA) facing the novel object were installed on opposite sides, allowing video recordings of the horses’ behavior during the entire test situation. The horses were habituated to the cameras through previous video recordings that were made during the horse’s daily routine in their stables and on pasture. The novel object test was completed precisely after 10 min, irrespective of the horses’ preceding behavioral responses. After each test, the test area was cleared of feces and uneven ground was leveled. If the test object was moved by the horse during the test, it was returned to its original position in the center of the test area. To minimize the influence of female pheromones on the behavior of the stallions, the stallions were tested before the mares on each of the test days.

### 2.3 Assessment of behavior during novel object test

Evaluation was performed by an independent observer who was experienced in equine behavior studies. The videos were randomized and blinded for the observation. The software “The Observer XT 16” (Noldus Information Technology, Wageningen, Netherlands, version 16.0.1203) was used to code the type, number and duration of certain behaviors according to a previously developed ethogram (Table 1). The ethogram was developed based on a previously defined standard ethogram but was specifically adapted for this study in order to enable a comprehensive and consistent evaluation of the horses’ behavior during the novel object test. All behaviors exhibited by the horses were included and each behavior was defined with precision. The time required for the horse to initially fixate, approach and touch the unknown object was observed, as well as the horse’s manner of interaction with the novel object. In addition to the video-based data collection, the horses were fitted with activity sensors (GT9XLink, ActiGraph, Pensacola, FL, USA). These sensors were attached to the left side of the horse’s neck using a collar and recorded relevant activities such as standing, walking, trotting, cantering and rolling (24). All horses were familiarized with wearing the sensor collars for several hours before the novel object tests. None of the horses showed any aversive behavior that was related to the attachment of the sensor collars.

**Table 1:** Ethogram for the observed behaviors during the novel object test.

Observed behavior	Parameter	Abbreviation	Description
Latency			
Latency time until the first fixation of the novel object	Duration (s)	Latency first fix obj	Horse is standing or moving, ears toward object, staring at object, object is in horse's binocular field of vision
Latency time until the first approach to the novel object	Duration (s)	Latency first approach obj	The object is in the horse's binocular field of vision, the horse looks at the object and takes one step toward the object (gait does not matter)
Latency time until the novel object is touched for the first time	Duration (s)	Latency first touch obj	A part of the horse's body touches the object intentionally (accidental tail flicking and touching the object does not count)
Interaction with the novel object			
Fixating the novel object	Duration (s)	Duration fix obj	Horse is standing or moving, ears toward object, staring at object, object is in horse's binocular field of vision, as soon as the horse changes the direction of gaze and then fixates the object again, this is counted as separate fixating events
	number (count)	nr fix obj	
Approaching the novel object	Number (count)	nr approach obj	The object is in the horse's binocular field of vision, the horse looks at the object and takes one step toward the object (gait does not matter), each first step after a standstill of > 2 s is counted as separate approaching events (subsequent steps without a standstill in between are counted as one event)
Sniffing on the novel object	Duration (s)	Duration sniff obj	Muzzle 20 cm away from the novel object, if there is no clear indication of the muzzle touching the object or simply sniffing the object, this behavior should be coded accordingly, as soon as the muzzle of the horse moves > 20 cm away from the object and then sniffs on the object again, this is counted as separate sniffing events
	number (count)	nr sniff obj	
Touching the novel object with the muzzle	Number (count)	nr touch obj muzzle	Touching with the muzzle, which leads to a visible movement of the object, as soon as the horses muzzle no longer touches the object for > 2 s and then touches the object again this is counted as separate touching events
Lifting the novel object	Number (count)	nr lift obj	Lifting the novel object from the ground using the muzzle
Touching the novel object with the forelimb	Number (count)	nr touch obj forelimb	Pawing or kicking with one front leg toward the object
Kicking the novel object with the hindlimb	Number (count)	nr kick obj	Kicking with hind leg toward the object, without necessarily touching it
Behaviors unrelated to the novel object			
Sniffing the ground	Duration (s)	Duration sniff ground	Horse is standing or moving with lowered head and sniffing on the ground (nose below the shoulder)
Chewing/licking	Number (count)	nr chewing	Movement with muzzle is visible
Grinning/flehming	Number (count)	nr flehming	Lifting the head up/forward with curling of the upper lip, looks like horse is smiling, incisors visible
Neighing/whinnying	Number (count)	nr neighing	Loud sound, most times while head is up, whole body is shaking
Squeaking	Number (count)	nr squeaking	High and loud sound
Blowing	Number (count)	nr blowing	Noisy exhalation, nostrils dilated, audibly increased, intermittent exhalation, forceful snorting
Snorting	Number (count)	nr snorting	Noisy exhalation, fluttering nostrils
Shaking head	Number (count)	nr headshaking	Shaking the head up and down, while standing or walking, aimed horizontal or vertical movement of the head
Tailswishing	Number (count)	nr tailswishing	Active moving tail suddenly from side to side, during standing or walking
Pawing	Number (count)	nr pawing	Arching action of horse's foreleg which strikes the ground
Stomping	number (count)	nr stomping	Suddenly flexing and then extending any limb to the ground
Lying down without rolling	Number (count)	nr lying down	Abdomen touches floor and all extremities flexed
Lying down with rolling	Number (count)	nr rolling	Rolling on the ground, starts when abdomen touchs the ground
Standing at the exit	Duration (s)	duration standing exit	Standing in the entrance/exit area, entire head is inside the imaginary triangle between object and door, as soon as the horse leaves the entrance/exit area, then moves back in this area and stands for > 2 sec, this is counted as separate events
	Number (count)	nr standing exit	
Defecating	Number (count)	nr defecating	
Urinating	Number (count)	nr urinating	
Rearing	Number (count)	nr rearing	The horse stands up on its hind legs with the forelegs off the ground
Bucking	Duration (s)	duration bucking	No defined gait, no clear rhythm recognizable, the horse can kick, jump, lowered head neck position, tail flap, head flap, round back, 2 or 4 legs off the ground at the same time
	Number (count)	nr bucking	
Yawning	Number (count)	nr yawning	Open the mouth wide, head often back, eye closed/ eye rolling for a few sec
Flinching	Number (count)	nr flinching	Sudden reflexive contraction of muscles, involuntary jerking movement with or without a single jump away from the object (more than one jump is bucking)
Shaking	Number (count)	nr shaking	e.g. after rolling
Rolling*	Duration (s)	Duration ROL	Rolling on the ground
Walking*	Duration (s)	Duration WAL	
Trotting*	Duration (s)	Duration TRO	
Galloping*	Duration (s)	Duration GAL	
Active*	Duration (s)	duration_ACT	Trot and gallop
Standing alert*	Duration (s)	duration SAL	Standing in a fixed and rigid position, head raised (poll above the withers), very little movement, attentively observing an object or sound

^*^Activities analyzed based on activity sensor data.

### 2.4 Statistical analysis

Due to technical issues, two video recordings and three sensor recordings from the novel object tests in the round pen could not be analyzed. As a result, data analysis included a total of 12 novel object tests in the paddock and 40 (video) / 39 (sensor) tests in the round pen. All statistical analyses were performed using R (25). The effect of test site (round pen or paddock), sex (mare or stallion) and auction (whether the horses participated in an auction or not) on the behavior during the novel object test was evaluated using linear or generalized linear mixed effects models with test site, sex and auction as well as the interaction between sex and test site and sex and auction as fixed effects and horse as random effect. The interaction between test site and auction was not included since the horses that had been tested twice in different test sites, had all been previously to an auction. By including horse as random effect, it was accounted for within-subject correlation in horses that were tested twice. Depending on the dependent variable being continuous (durations) or discrete (count data), linear or negative binomial generalized linear mixed effects models were fitted using the ‘lmer’ or ‘glmer.nb’ function in R. The significance of the fixed effects on the behavior during the novel object test was evaluated by comparing full models to models without the fixed effects by means of likelihood ratio tests using the ‘anova’ function in R. Normality of the error distributions of the linear regression models was evaluated by means of normal probability plots of the residuals. The results are presented as box plots and estimated marginal means. Results that are presented in Table 2 are provided as estimated marginal means ± standard error. In order to describe the behavior of the horses during the novel object tests as comprehensively as possible, a relatively large number of different behavioral parameters was recorded during this study. To reduce the dimensionality of the data and to find associations between different behavioral parameters, principal component analysis was conducted using the ‘prcomp’ function in R. Subsequently, a kmeans cluster analysis of the coordinates of the different variables on the principal components (retaining only those components with an eigenvalue >1) was carried out. The resulting clusters were visualized in the loadings plot of the first two principal components.

**Table 2:** Statistical analysis of the behavior during the novel object test (results are presented as estimated marginal means ± standard error).

	Paddock	Round pen	Mare	Stallion	Auction	No auction
Observed behavior (Abbrev.)						
Latency						
Latency first fix obj (s)	2.7 ± 1.1*	6.9 ± 1.3*	3.4 ± 1.1	5.6 ± 1.4	7.0 ± 1.4*	2.7 ± 1.0*
Latency first approach obj (s)	47.9 ± 9.6**	11.5 ± 4.8**	11.7 ± 3.9*	47.3 ± 13.3*	23.7 ± 4.9	23.2 ± 9.1
Latency first touch obj (s)	320 ± 83	386 ± 50	346 ± 65	358 ± 67	307 ± 41	403 ± 102
Interaction with the novel object						
Duration fix obj (s)	3.0 ± 4.7*	14.2 ± 2.3*	7.0 ± 3.4	10.2 ± 3.3	7.2 ± 2.4	10.1 ± 4.5
nr fix obj (count)	1.2 ± 0.0044*	5.7 ± 0.027*	2.1 ± 0.0077	3.2 ± 0.015	2.5 ± 0.0092	2.7 ± 0.013
nr approach obj (count)	6.2 ± 0.93***	10.1 ± 0.79***	8.7 ± 1.0	7.2 ± 0.85	7.8 ± 0.74	8.0 ± 1.2
Duration sniff obj (s)	5.3 ± 1.9	5.0 ± 1.0	5.6 ± 1.5	4.7 ± 1.5	5.0 ± 1.1	5.3 ± 1.9
nr sniff obj (count)	1.5 ± 0.46	1.6 ± 0.35	1.6 ± 0.45	1.5 ± 0.44	1.9 ± 0.41	1.2 ± 0.47
nr touch obj muzzle (count)	0.17 ± 0.10***	1.02 ± 0.44***	0.38 ± 0.22	0.46 ± 0.26	0.67 ± 0.29	0.27 ± 0.19
nr touch obj forelimb (count)	0.060 ± 0.062	0.14 ± 0.095	0.098 ± 0.080	0.088 ± 0.070	0.061 ± 0.045	0.14 ± 0.13
nr kick obj (count)	2.8 ± 1.1	1.4 ± 0.33	2.3 ± 0.68	1.7 ± 0.56	2.8 ± 0.58	1.4 ± 0.62
Behaviors unrelated to the novel object						
Duration sniff ground (s)	196 ± 21	187 ± 13	160 ± 18**	224 ± 18**	219 ± 15*	164 ± 23*
nr chewing (count)	1.0 ± 0.38*	0.50 ± 0.16*	0.47 ± 0.19	1.1 ± 0.41	1.2 ± 0.33	0.43 ± 0.23
nr neighing (count)	1.9 ± 0.78	2.5 ± 0.95	5.2 ± 2.4**	0.93 ± 0.51**	1.6 ± 0.70	3.0 ± 1.7
nr squeaking (count)	0.92 ± 0.45*	0.36 ± 0.14*	0.78 ± 0.34	0.43 ± 0.21	0.43 ± 0.16	0.78 ± 0.45
nr blowing (count)	1.9 ± 0.46***	33.8 ± 4.8***	10.4 ± 2.3*	6.1 ± 1.3*	7.4 ± 1.4	8.5 ± 2.2
nr snorting (count)	1.6 ± 0.52	1.2 ± 0.31	1.1 ± 0.38	1.7 ± 0.58	1.8 ± 0.48	1.0 ± 0.46
nr headshaking (count)	7.9 ± 2.0	2.7 ± 0.60	5.7 ± 1.6	3.8 ± 1.1	3.6 ± 0.82	6.0 ± 2.1
nr tailswishing (count)	0.35 ± 0.31	0.72 ± 0.32	0.57 ± 0.34	0.44 ± 0.31	0.48 ± 0.26	0.53 ± 0.41
nr pawing (count)	5.9 ± 3.6	5.0 ± 2.3	2.8 ± 1.6*	10.4 ± 4.8*	6.2 ± 2.7	4.7 ± 3.0
nr rolling (count)	0.066 ± 0.055	0.10 ± 0.072	0.030 ± 0.027*	0.23 ± 0.17*	0.15 ± 0.092	0.047 ± 0.049
Duration standing exit (s)	91.7 ± 25.9	86.2 ± 12.6	90.3 ± 18.9	87.6 ± 18.4	77.2 ± 13.4	100.7 ± 24.7
nr standing exit (count)	10.4 ± 1.7***	6.5 ± 0.73***	10.6 ± 1.6*	6.4 ± 1.1*	5.9 ± 0.77**	11.5 ± 2.2**
nr defecating (count)	0.36 ± 0.16***	1.8 ± 0.23***	0.99 ± 0.26	0.65 ± 0.18	0.74 ± 0.18	0.88 ± 0.27
Duration bucking (s)	18.0 ± 3.9	11.1 ± 1.9	14.0 ± 2.9	15.1 ± 2.8	16.7 ± 2.0	12.4 ± 3.7
nr bucking (count)	6.7 ± 1.3	3.5 ± 0.49	5.2 ± 0.94	4.5 ± 0.82	5.6 ± 0.75	4.1 ± 1.0
nr flinching (count)	2.8 ± 0.86*	1.4 ± 0.28*	1.8 ± 0.51	2.1 ± 0.55	1.4 ± 0.31	2.6 ± 0.89
nr shaking (count)	1.4 ± 0.60***	0.32 ± 0.11***	0.45 ± 0.19	1.0 ± 0.38	0.60 ± 0.20	0.77 ± 0.40
Duration ROL (s)	7.0 ± 3.5	2.4 ± 1.8	1.8 ± 2.6	7.6 ± 2.7	5.5 ± 2.0	3.9 ± 3.4
Duration WAL (s)	89.2 ± 16.1*	127.6 ± 9.0*	105.3 ± 12.8	111.6 ± 13.7	106.9 ± 10.5	109.9 ± 16.4
Duration TRO (s)	79.4 ± 16.5	70.0 ± 10.9	98.5 ± 15.0*	50.8 ± 16.4*	71.5 ± 12.9	77.9 ± 18.9
Duration GAL (s)	28.7 ± 6.4*	12.8 ± 3.3*	26.4 ± 4.8	15.1 ± 5.1	18.4 ± 3.8	23.0 ± 6.2
Duration ACT (s)	109.5 ± 20.8	82.6 ± 12.8	125.2 ± 17.8*	67.0 ± 19.4*	90.4 ± 15.1	101.8 ± 22.6
Duration SAL (s)	25.5 ± 11.4*	57.8 ± 6.7*	41.5 ± 9.4	41.8 ± 10.1	47.0 ± 7.8	36.3 ± 11.9

Asterisks indicate different levels of significance: *p < 0.05, **p < 0.01, ***p < 0.001.

## 3 Results

**Figure 3:**
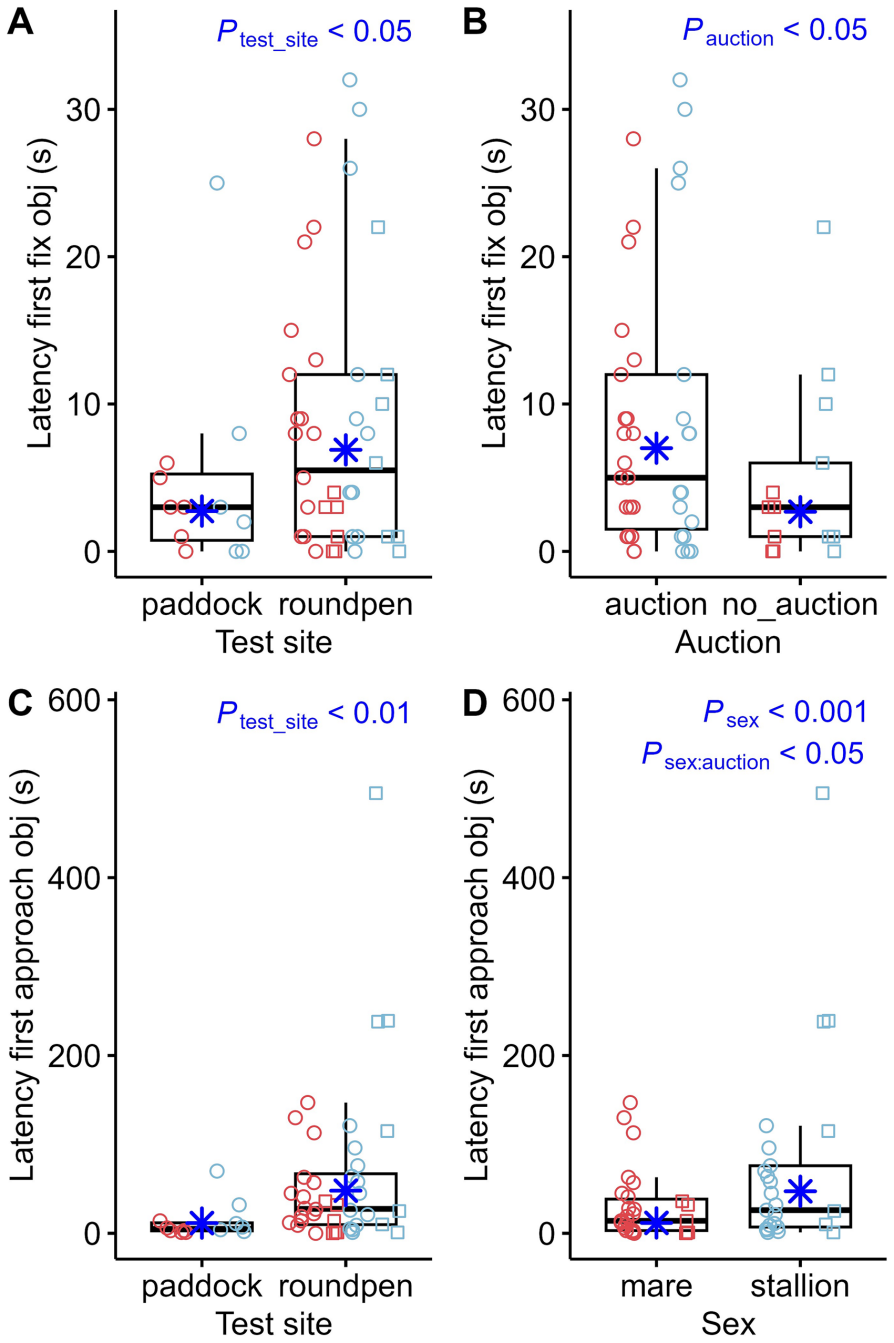
Latency time until the first fixation of the object (Latency first fix obj) compared between test sites (**A**; paddock: *n* = 12, round pen: *n* = 40) and between horses that participated in an auction (*n* = 39) or not (*n* = 13) **(B)** as well as latency time until the first approach toward the object (Latency first approach obj) compared between test sites **(C)** and sexes **(D)**. The box plots display the distribution of data by indicating the median and the interquartile range (IQR). Individual data points from mares (*n* = 27) are shown either as red circles (horses that participated in an auction, *n* = 21) or red squares (horses that did not participate in an auction, *n* = 6). Individual data points from stallions (*n* = 25) are shown in blue circles (*n* = 18) or squares (*n* = 7) accordingly. The marginal means estimated by the mixed effects models are shown as blue asterisks. *p*-values result from negative-binomial regression models.

**Figure 4:**
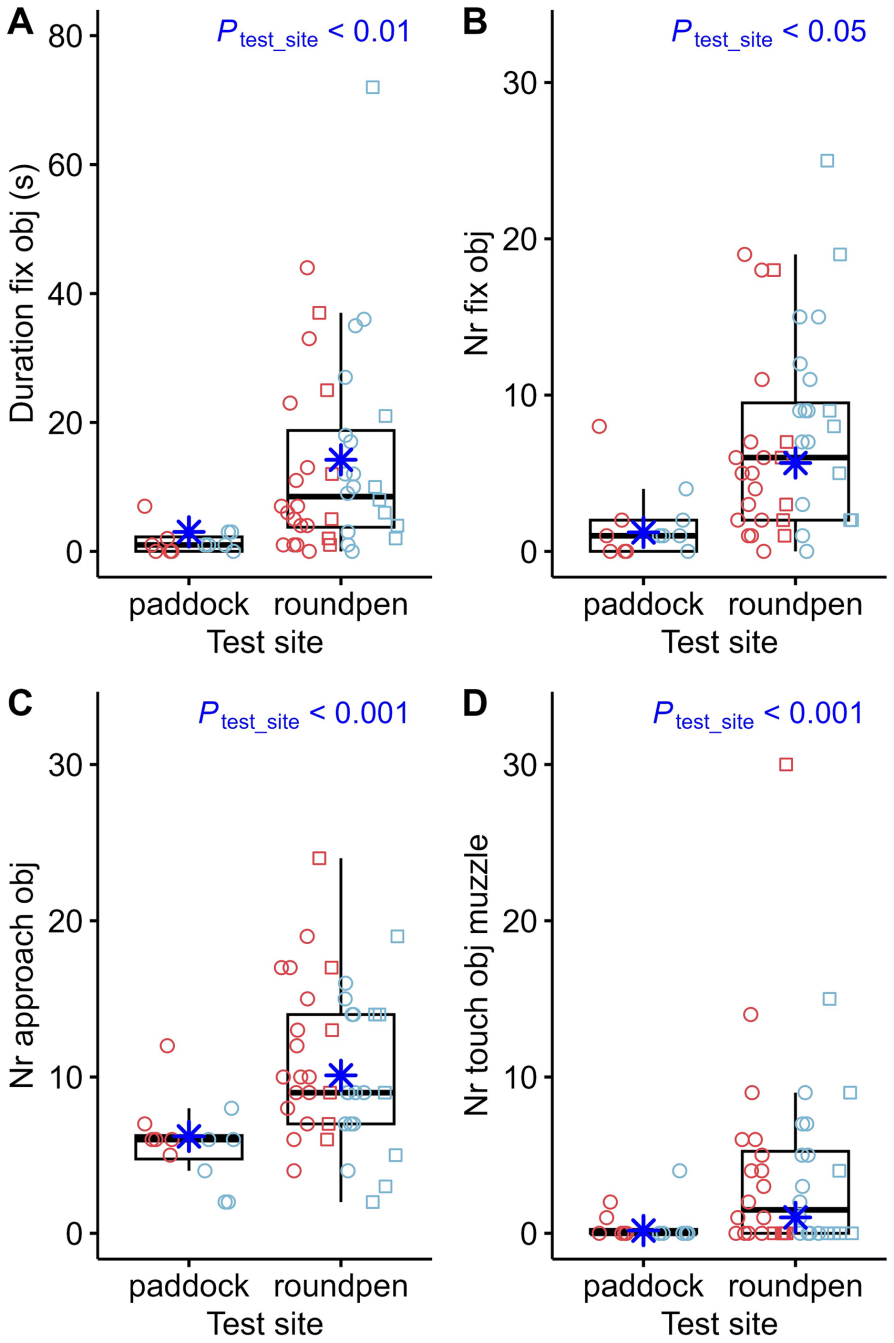
Duration of fixation of the object (Duration fix obj) **(A)**, number of fixations of the object (Nr fix obj) **(B)**, number of approaches toward the object (Nr approach obj) **(C)** and number of touches of the object with the muzzle (Nr touch obj muzzle) **(D)** compared between test sites (paddock: *n* = 12, round pen: *n* = 40). The box plots display the distribution of data by indicating the median and the interquartile range (IQR). Individual data points from mares (*n* = 27) are shown either as red circles (horses that participated in an auction, *n* = 21) or red squares (horses that did not participate in an auction, *n* = 6). Individual data points from stallions (*n* = 25) are shown in blue circles (*n* = 18) or squares (*n* = 7) accordingly. The marginal means estimated by the mixed effects models are shown as blue asterisks. *p*-values result from linear regression models for durations and negative-binomial regression models for count data.

**Figure 5:**
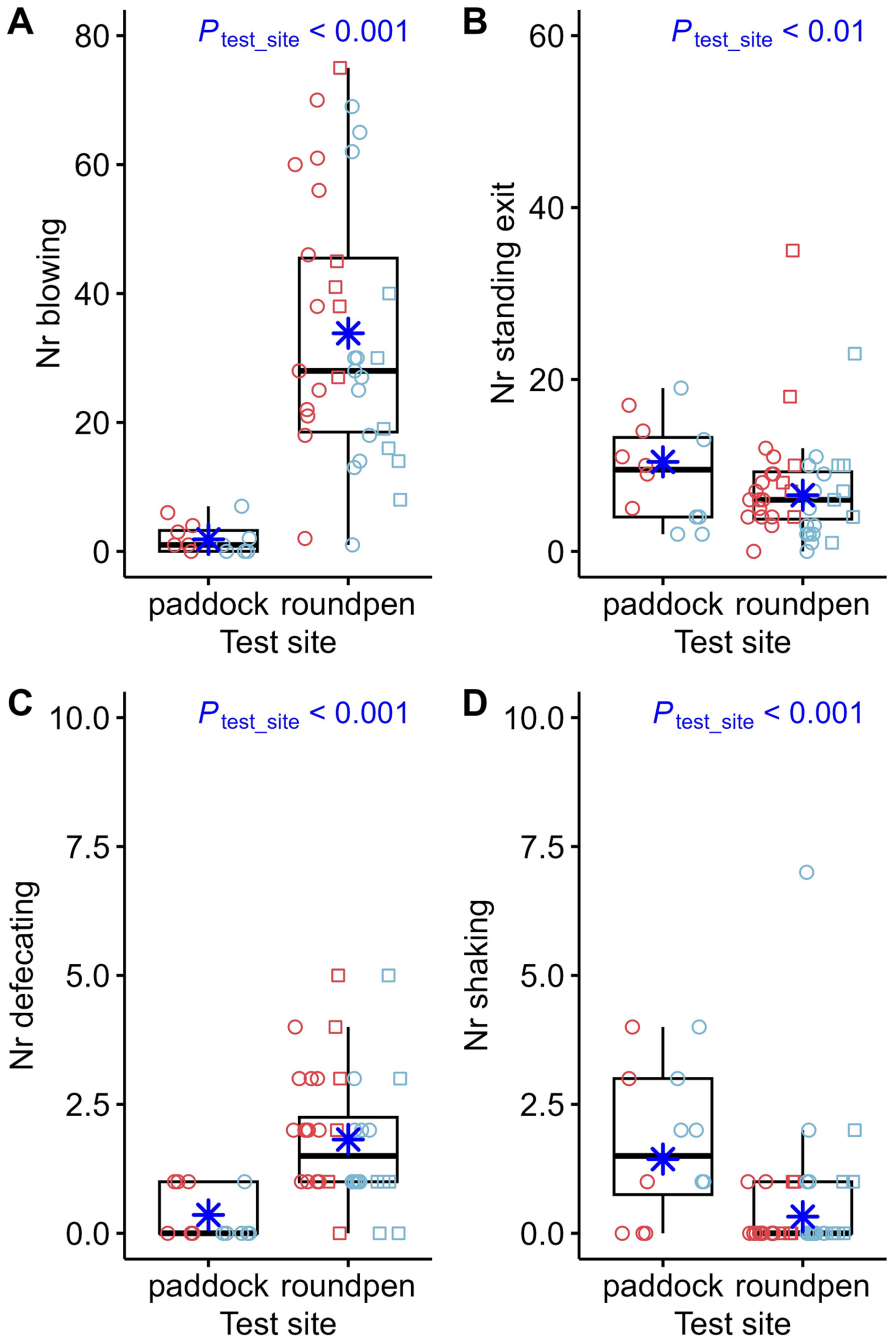
Number of blowing (Nr blowing) **(A)**, number of standing at the exit (Nr standing exit) **(B)** number of defecations (Nr defecating) **(C)** and number of shaking (Nr shaking) **(D)** compared between test sites (paddock: *n* = 12, round pen: *n* = 40). The box plots display the distribution of data by indicating the median and the interquartile range (IQR). Individual data points from mares are shown either as red circles (horses that participated in an auction, *n* = 21) or red squares (horses that did not participate in an auction, *n* = 6). Individual data points from stallions are shown in blue circles (*n* = 18) or squares (*n* = 7) accordingly. The marginal means estimated by the mixed effects models are shown as blue asterisks. *p*-values result from negative-binomial regression models.

**Figure 6:**
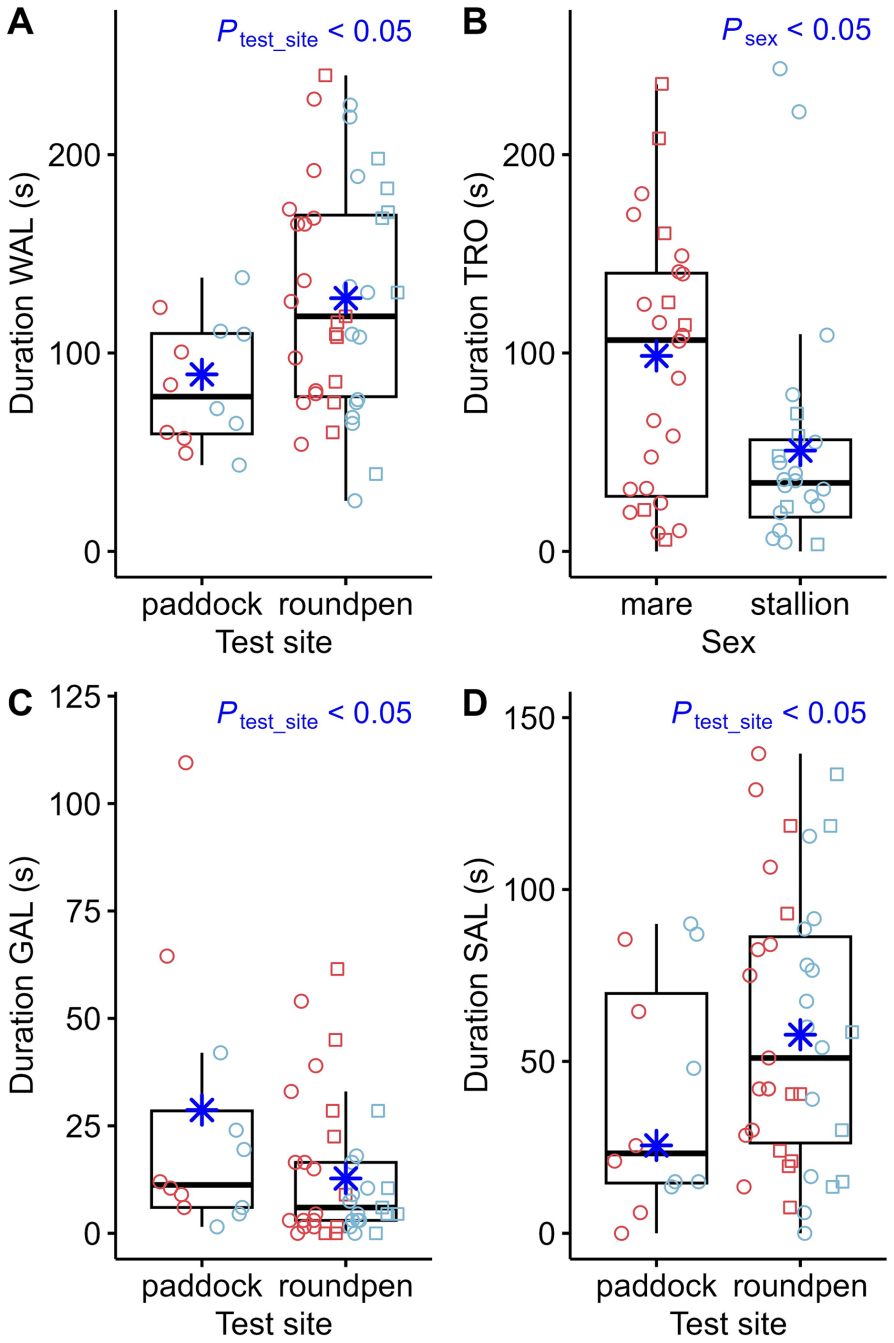
Duration walking (Duration WAL) during the novel object test compared between test sites (paddock: *n* = 12, round pen: *n* = 39) **(A)**, duration trotting (Duration TRO) compared between sexes **(B)** as well as duration galloping (Duration GAL) **(C)** and standing alert (Duration SAL) **(D)** compared between test sites. The box plots display the distribution of data by indicating the median and the interquartile range (IQR). Individual data points from mares (*n* = 27) are shown either as red circles (horses that participated in an auction, *n* = 19) or red squares (horses that did not participate in an auction, *n* = 8). Individual data points from stallions (*n* = 24) are shown in blue circles (*n* = 18) or squares (*n* = 6) accordingly. The marginal means estimated by the mixed effects models are shown as blue asterisks. *p*-values result from linear regression models for durations and negative-binomial regression models for count data.

The most significant differences in behavior during the novel object test were related to the impact of the test site. When comparing the behavior of the 12 horses that completed the novel object test on both test sites, the following observations can be made: 11 out of 12 horses had touched the novel object in the paddock. However, 4 of these 12 horses did not touch the object in the round pen. Comparison of the effect of test sites showed multiple significant differences: The latency time for the first fixation on the object (Figure 3A) and the first approach (Figure 3C) was higher in the round pen. However, the effects of test site, sex and whether the horses had attended an auction on the latency time to touch the object for the first time were not statistically significant. When the novel object test was performed in the round pen, the horses fixated the novel object longer (Figure 4A). The number of fixations on the object (Figure 4B), approaches to the object (Figure 4C) and touches of the object with the muzzle (Figure 4D) were higher in the round pen. With regard to those behaviors that are not directly related to the novel object, blowing (Figure 5A) and defecating (Figure 5C) were more frequent in the round pen than in the paddock. On the other hand, the behaviors chewing and licking, squeaking, standing at the exit (Figure 5B), flinching and shaking (Figure 5D) were more frequent in the paddock than in the round pen. Depending on the test site, activity sensor data indicated that the horses spent more time walking (Figure 6A) and less time galloping (Figure 6C) in the round pen than in the paddock. Moreover, the horses spent more time standing alert (Figure 6D) in the round pen than in the paddock.

**Figure 7:**
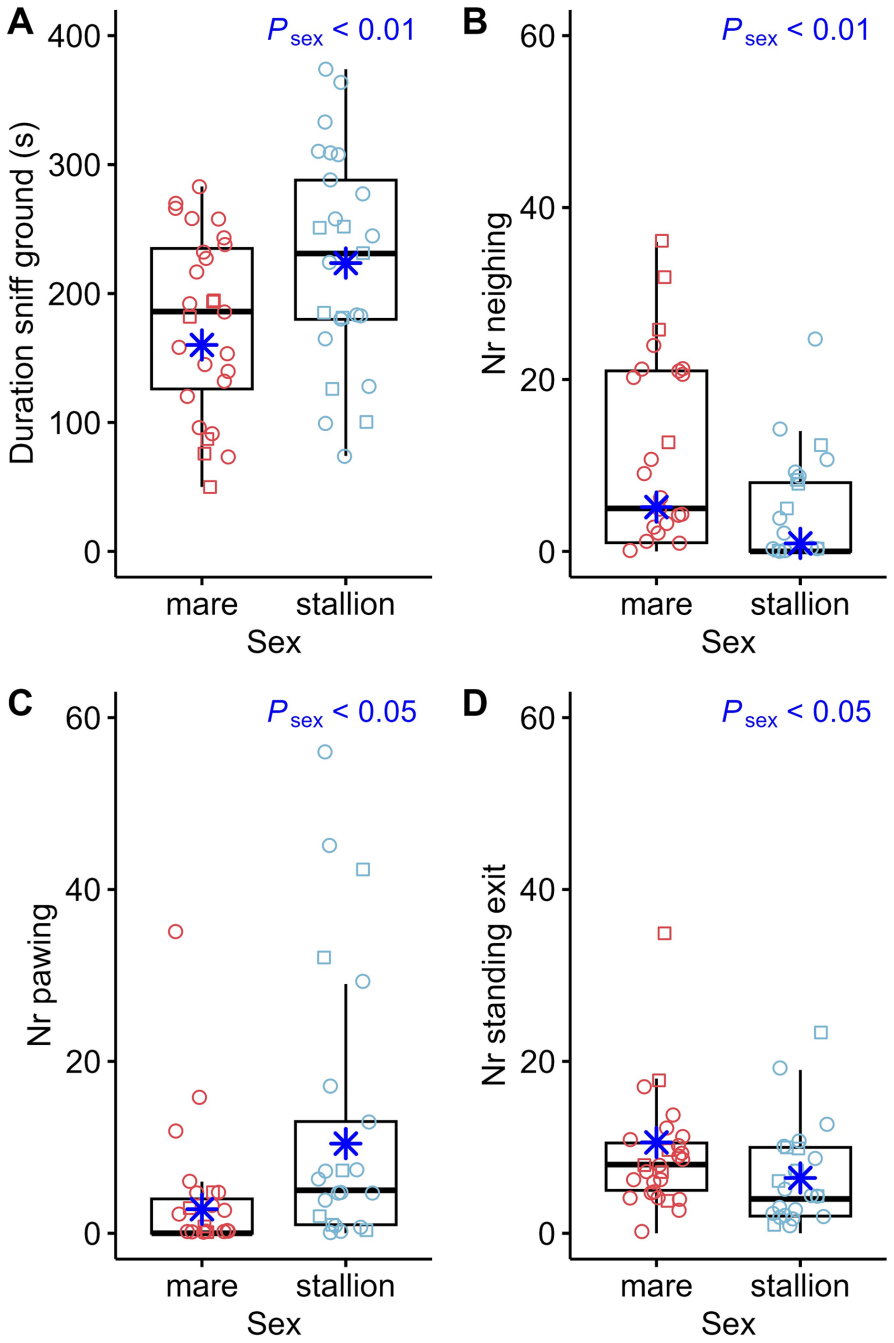
Duration of sniffing the ground (Duration sniff ground) **(A)**, Number of vocalizations (Nr neighing) **(B)**, number of pawing (Nr pawing) **(C)** and number of standing at the exit (Nr standing exit) **(D)** compared between sexes (stallions: *n* = 25, mares: *n* = 27). The box plots display the distribution of data by indicating the median and the interquartile range (IQR). Individual data points from mares are shown either as red circles (horses that participated in an auction, *n* = 21) or red squares (horses that did not participate in an auction, *n* = 6). Individual data points from stallions are shown in blue circles (*n* = 18) or squares (*n* = 7) accordingly. The marginal means estimated by the mixed effects models are shown as blue asterisks. *p*-values result from linear regression models for durations and negative-binomial regression models for count data.

Some behavioral parameters also appeared to be significantly associated with sex. Mares approached the novel object for the first time (Figure 3D) earlier than stallions. Stallions sniffed the ground (Figure 7A) longer and pawed (Figure 7C) more frequently than mares. Vocalization (Figure 7B) and standing at the exit (Figure 7D) were more striking in mares. Mares also appeared to be more active, with longer durations trotting (Figure 6B) than stallions.

**Figure 8:**
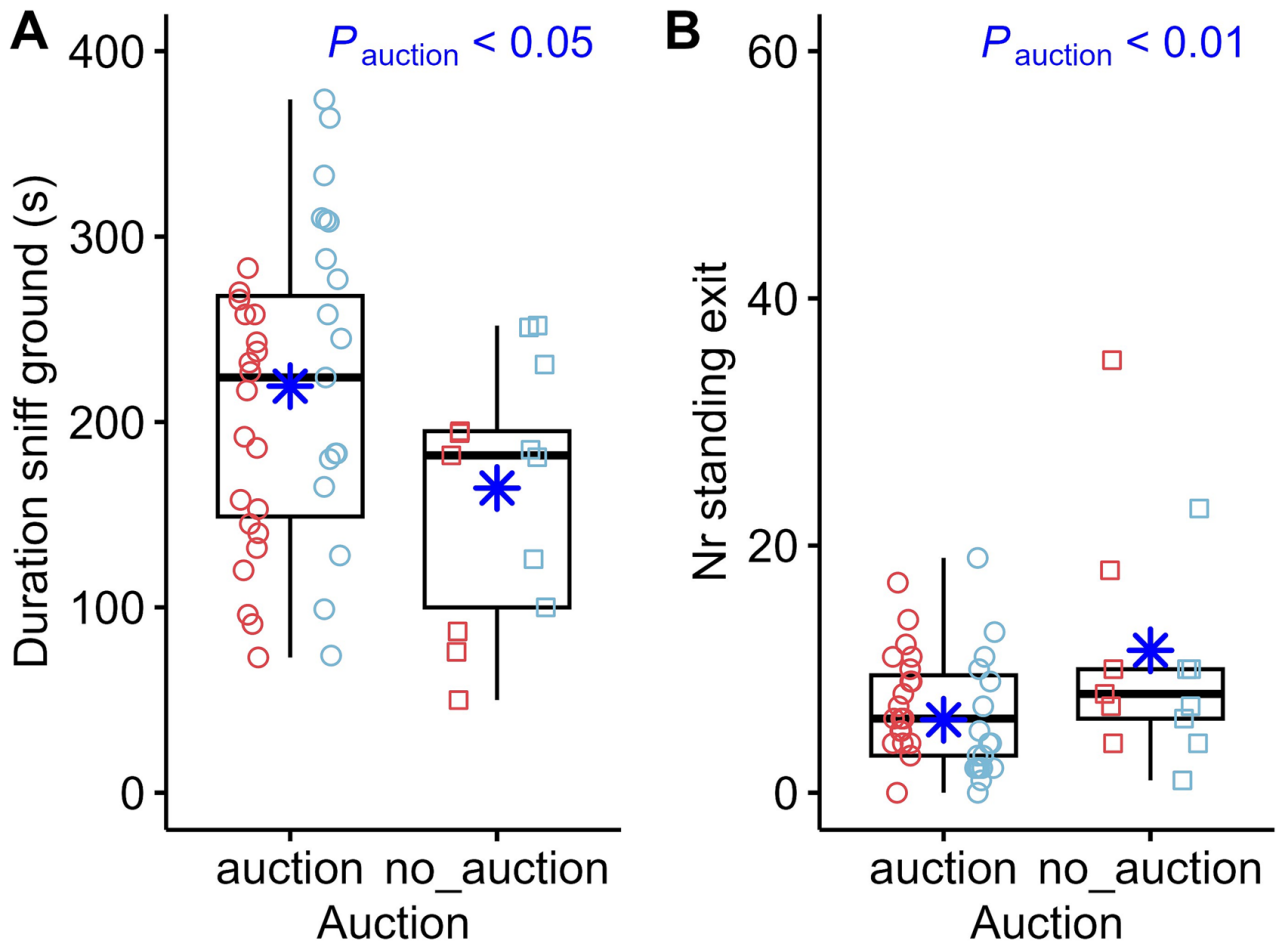
Duration of sniffing the ground (Duration sniff ground) **(A)** and number of standing at the exit (Nr standing exit) **(B)** compared between horses that participated in an auction (*n* = 39) or not (*n* = 13). The box plots display the distribution of data by indicating the median and the interquartile range (IQR). Individual data points from mares are shown either as red circles (horses that participated in an auction, *n* = 21) or red squares (horses that did not participate in an auction, *n* = 6). Individual data points from stallions are shown in blue circles (*n* = 18) or squares (*n* = 7) accordingly. The marginal means estimated by the mixed effects models are shown as blue asterisks. *p*-values result from linear regression models for durations and negative-binomial regression models for count data.

When comparing whether or not the horses had participated in an auction, the following significant results could be demonstrated: The latency time until the first fixation of the object (Figure 3B) as well as the duration of sniffing the ground (Figure 8A) was longer for the horses that attended an auction. Furthermore, the horses that did not participate in an auction were standing at the exit (Figure 8B) more often.

**Figure 9:**
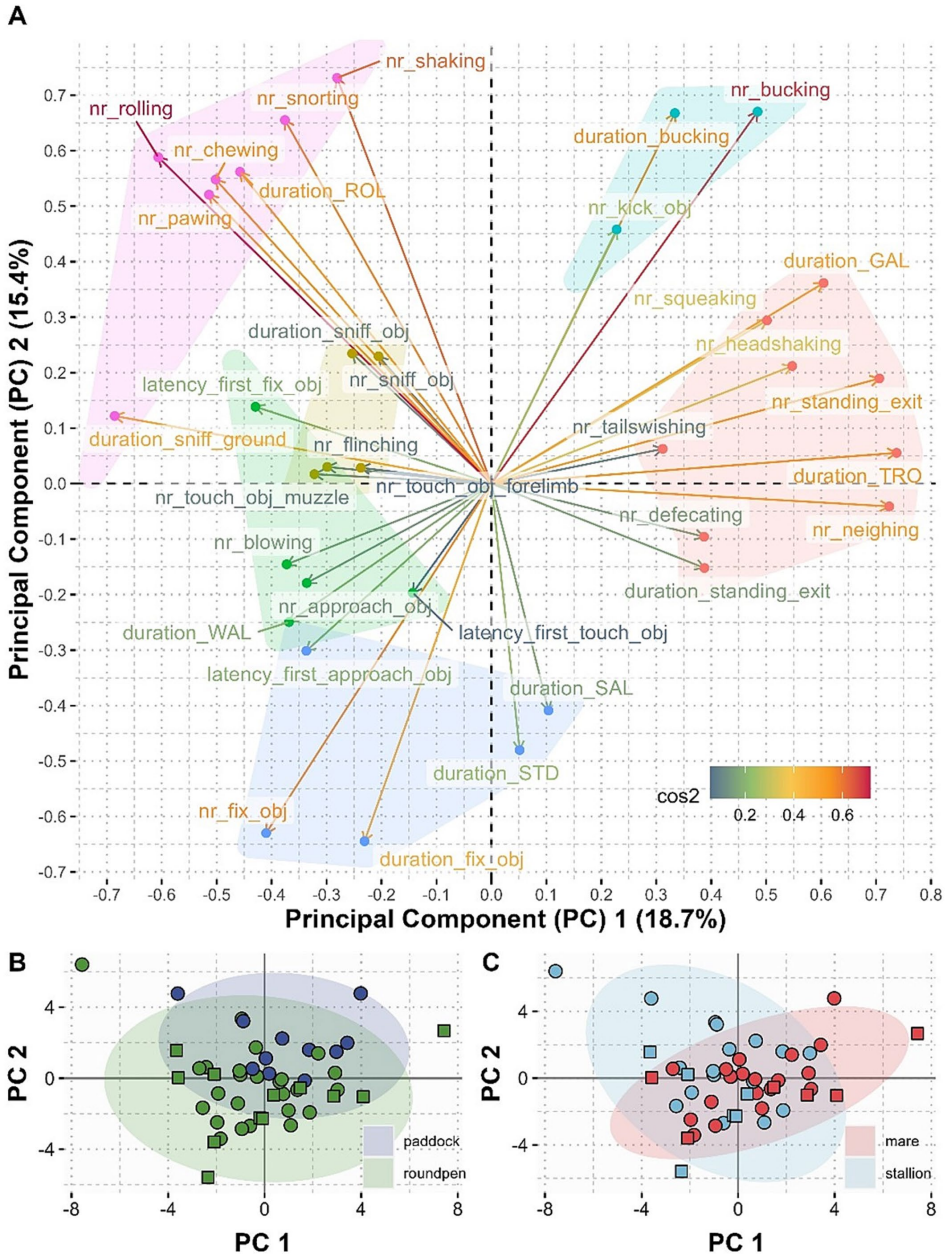
Correlation (loadings) between the different behavioral variables and the first (PC 1) and second principal component (PC 2) **(A)**. The variation explained by the principal components is given in % of total variability in the axe’s labels. The quality of representation of the variables by the principal components (cos2) is indicated by the color of the arrows and labels. The further away a variable is from the origin of the plot, the more important it is to interpret these components. The closer to the center of the plot a variable is, the less important it is for the first two components. Variables whose arrows point in the same direction are positively correlated and variables whose arrows point in opposite directions are negatively correlated to each other. The clusters identified by means of kmeans clustering of the coordinates of the variables on the first 9 principal components (eigenvalue >1) are indicated by different coloring of the data points associated with the variables. Individual data points for PC 1 and PC 2 compared between novel object tests conducted in the round pen (green) or paddock (dark blue) **(B)** and compared between mares (red) and stallions (light blue) **(C)**. Individual data points form horses that participated in an auction are shown as circles, horses that did not participate in an auction are shown as squares **(B,C)**.

Cluster analysis was performed using the first 9 principal components accounting for 77% of the variation in the data. The resulting clusters are visualized in the loadings plot of the first two principal components (Figure 9A). The principal component and k means cluster analysis show that certain behavioral parameters that have been observed during the novel object test were more associated with each other than others and formed clusters of parameters that jointly contribute to the principal components. Six main clusters of highly associated behaviors could be identified and are visualized in Figure 9A. When comparing the individual data points between test sites (Figure 9B) and sexes (Figure 9C), it becomes apparent that the point cloud for the tests conducted on the paddock is slightly shifted toward the upper left quadrant of the plot, indicating that the horses exhibited less engagement with the novel object. The point cloud for the mares is slightly shifted toward active and anxious behavior, whereas the point cloud for stallions is slightly shifted toward exploratory and comfort behavior. However, both point clouds overlap in the region of behaviors related to the novel object.

## 4 Discussion

The results of this study show considerable inter-individual differences in the behavior of horses during the novel object test. Moreover, the behavior of the horses was significantly influenced by the external circumstances of the test situation since it could be shown that the test site had a significant effect on the behavior of the horses. The horses fixated the novel object faster and made the first approach toward the object earlier in the paddock than in the round pen. On the other hand, the total duration for which the horses fixated the object was shorter and the number of fixations as well as the number of approaches toward the object and touches of the object with the muzzle was lower in the paddock. Thus, the horses engaged with the object earlier but for a shorter time when the test took place in the paddock. Little interaction with the novel object may indicate fear or stress (7). On the other hand, exploration of the novel object, such as sniffing or nibbling, is evaluated as absence of anxiety (26). The difference in these behaviors between test sites may indicate that in the environment that is more open, the horses are less afraid to approach the novel object but are too distracted by the surroundings to explore the object more closely. However, the faster fixation and approach toward the novel object and the lower extent of explorative behavior toward the object may also be attributed to a habituation effect from the first test although the object and location was different. In view of the influence of the testing environment of the behavior during the novel object test, the question arises to what extent the influence of the test conditions was currently taken into account in the interpretation of the results of novel object tests. The German Veterinary Society recommends including a novel object test in the ‘Examination protocol for racehorses before the start of training and first start of life’. Although it is recommended that the test should be performed in a familiar, enclosed area that is preferably covered, test environments will vary depending on the local conditions of the stable. The test environments evaluated in this study were chosen to reflect different conditions that can be typically found in stables accommodating racehorses. It is also recommended that the tests prior to the start of training and the horse’s first race should be performed on the same test site on both occasions to ensure comparability. The results of our study support this recommendation as they demonstrated that the same horses exhibited significantly different behavior when tested in different environments. Moreover, the protocol recommends a maximum test duration of 5 min. The latency for the horse to olfactory explore the novel object for the first time, approach it and touch it is measured but no further interpretation of the behavioral test results is provided here (9).

The recommendation to perform the novel object test at the same location both times is supported, but the question of feasibility arises. Many horses are no longer housed in the same barn before the first training session and later before the first race, making it difficult to compare data. Given the findings of this study, it may be worthwhile to consider whether the recommended 5-min test time is sufficient, as the average latency time until the horses touched the object for the first time was higher than 5 min.

To ensure a consistent interpretation of the results, it is crucial to consider the test location. Comparing results obtained in a paddock with those from a round pen is not appropriate. As horses are prey species and highly sensitive to their environment, any external stimulus can affect the results of the novel object test. Therefore, it is recommended to compare the results of novel object tests only within individuals under the same conditions. The presence of humans also influences the horse’s behavior, which is why indirect behavioral observation using cameras should be chosen.

The study did not sufficiently investigate the extent to which familiarity with the test site affects the results. If feasible, it is advisable to acclimatize the test location to relate the animals’ reaction to the novel object rather than the unfamiliar environment (19). The horses in the study were familiar with the test sites but they were not familiar with being kept alone in the round pen or paddock. Horses are obligate social creatures, and social separation can be a significant stressor that affects their behavior (27). Additionally, habituation to the test site can be considered as training for social separation (13). On the other hand, previous experiences with the test environments should also be considered. Horses that experienced stressful situations in the same or similar environment, for example during training, may also exhibit higher stress levels during the novel object test. In summary, the location of the test significantly influences the results of the novel object test. It is unclear whether these differences in results are due to environmental factors such as increased visual, olfactory, and acoustic stimuli or exposure to weather conditions on the paddock, or if the differences are due to habituation to the test areas. As this study clearly indicates that different test environments lead to different results of novel object tests, one of the key issues when planning experiments involving behavioral tests is an appropriate choice of the test environment.

The novel object test is a behavioral assessment that measures escape and exploratory behavior which are not primarily sex-specific (2). The studies by Wolff et al. (19) and Lesimple et al. (18) were unable to assign any further significance to age and sex in the novel object test. When considering the sex of the horses, it is also important to take into account their age and sexual maturity. The horses in this study were between 23 and 27 months old, which suggests that they have reached sexual maturity, as it typically occurs at an age of 12–20 months (28). Our study found no sex-specific differences in the direct interaction of the horses with the novel object with the exception that mares tended to approach the novel object significantly earlier than stallions. However, since we used a very comprehensive ethogram, we also observed many individual behaviors that were not directly related to the novel object. Some of these behaviors appear to be influenced by sex. It could be shown that regardless of the test site, stallions sniff the ground longer than mares. Sniffing the ground can be classified as a form of exploratory behavior, which allows horses to gain new experiences and learn independently (28). Moreover, sniffing is a behavior that is especially used by stallions to gain information about other horses. Pawing was also more frequently observed in stallions than in mares, which also seems to be related to a more pronounced exploratory behavior in stallions compared to mares. Moreover, it is evident that the mares were more active during the novel object test, exhibiting a markedly higher duration of trotting than the stallions. Visser et al. (29) demonstrated that horses kept in pairs trotted and galloped more during a novel object test than horses kept individually. Increased activity can also be observed as a reaction of the horses to social isolation (30). Compared to stallions, the behavior of mares seems to be more strongly characterized by the effect of social isolation which is also indicated by the higher number of vocalizations and higher occurrence of standing at the exit. Increased vocalization has been associated with anxious behavior and may be an indicator of stress (7). The sex-specificity of behaviors associated with exploration of the environment and social isolation must be interpreted in relation to the natural behavior of horses. Stallions usually leave their natal herd at the age of approximately 2 years and may therefore be less dependent on their strong social bonds (19).

The findings of this study suggest that participation in an auction may have some influence, albeit limited. It was observed that horses that had participated in an auction exhibited a longer latency period until the first fixation of the object and spent more time sniffing the ground. However, further research is necessary to clarify the significance of these results. It appears that the horses that had participated in the auction may have been more accustomed to different visual stimuli. Nevertheless, there were no significant differences in terms of latency to first approach and touch the object, and no differences in direct interactions with the novel object between the groups. However, horses that did not have participated in an auction tended to stand longer close to the exit, which may be a reaction to social isolation. The horses investigated in this study were all about the same age but had different previous experiences probably resulting in different degrees of habituation to unknown situations. According to Visser et al. (17), it was observed that horses tend to exhibit less reactivity confronting unfamiliar objects as they age. The study also found that older horses approach the object more quickly, spent less time galloping and trotting, and more time exploring their surroundings. Further investigations could explore whether these results are solely attributed to the age of the animals or if previous experiences, such as participation in an auction or exposure to different housing or training conditions, may have an influence. A 2018 study examined the rearing conditions of foals, with a particular focus on the weaning process from the mare, and found that these conditions may affect the behavioral development of the horses, which in turn may indirectly affect the outcomes of a novel object test (31).

In view of the complexity of the horse’s behavior in response to unfamiliar situations, the question arises whether the interpretation of novel object tests on the basis of single isolated parameters is adequate. Thus, it would be desirable to reduce the number of parameters to be evaluated without losing information. One way to tackle this problem could be to systematically aggregate several small-scale behavioral parameters into fewer functional groups based on their correlation and joint contribution in explaining the observed variability by means of principal component analysis and clustering. Principal component analysis provides a low dimensional representation of the high dimensional data set of behavioral parameters collected within this study and was primarily intended to give a visual approximation of the systematic information contained in the multivariate data. It has to be noted, however, that this visual approximation can only be partial as scatter plots take only two dimensions into account, and hence only explain a part of the variability of the data. Unless the information in the data is truly contained in two or three dimensions, most visualizations will only give a limited view of the multivariate phenomenon. This study identified six main clusters of highly associated behaviors that are also functionally related. The cluster, including behaviors like increased activity (trotting and galloping), vocalization, defecating, tailswishing, standing at the exit etc., which can most likely be associated with fear and stress (30, 32) is very well separated from the clusters including those behaviors that are related to the exploration of the novel object and the direct test environment. Thus, horses exhibiting signs of high levels of excitement tend to pay less attention to the novel object or the direct testing environment. This supports the assumption that low engagement with the novel object indicates fear or stress and high engagement with the object indicates the absence of fear (26). It could also be demonstrated a clear separation between the duration and number of times the horses were sniffing at or touching the novel object with the muzzle or forelimb and the number and duration of fixations. This observation emphasizes the importance of distinguishing between the different levels of engagement with the novel object. Horses that spend considerable time looking at the novel object and maybe even approach it by taking some steps toward it do not necessarily explore the novel object by sniffing and touching it with the muzzle or forelimb. Moreover, kicking toward the novel object was associated with active behavior, especially bucking, but clearly separated from the behaviors describing a curious exploration of the object. Thus, this kind of behavior may be interpreted as defensive behavior when confronting the object and seems more associated with anxious behavior. Although this observation cannot be substantiated by literature that shows a clear association between kicking behavior and anxious or defensive behavior. On the other hand, behaviors that are related to the exploration of the environment (e.g., sniffing the ground and pawing) and comfort behavior like rolling are positively correlated with the exploration of the novel object. Thus, this further supports the assumption that horses exhibiting those behaviors seem to be less anxious.

In order to transfer the data from this study to the entire population of thoroughbred horses, it would be beneficial to increase the number of animals involved. Furthermore, the sample of horses utilized in this study encompasses a range of variability within the horse population. This includes horses of diverse pedigrees, early and late maturing horses, and horses of different sexes. One limitation of the study is that only one age group of horses was examined. It is also noteworthy that only 12 horses were tested in both the round pen and the paddock. The study design was deliberately chosen to determine which differences in behavior were due to the test location and not to inter-individual differences between the horses. The order in which the novel object test was performed should also be considered. For practical reasons, the 12 horses were all tested in the round pen first, and the second novel object test was conducted in the paddock. Because the individual novel object tests differed in the objects used, it is not possible to conclusively determine how the subjective perception of the objects influenced the horses’ behavior during the test. It would have been beneficial to test half of the horses with one object and the other half with the other object in order to avoid the effect different objects might have on the behavior of the horses during the test. Another limitation of this study is that only one independent observer evaluated the behavior during the novel object tests. Interrater reliability could therefore not be obtained.

## 5 Conclusion

In conclusion, it may be advantageous to establish a standardized method for executing and interpreting novel object tests with horses. The test should be performed in a calm environment to minimize external influences. It is recommended to use a round pen with visual barriers, as horses are highly sensitive prey animals and may be easily distracted by external factors. The timing of the test should also be taken into consideration. To promote accurate results, it is suggested to choose a time frame with expected minimal interference with other horses, personnel, or agricultural machinery and a duration extending 5 min. For consistency, it is crucial to perform the novel object test at the same location especially if several tests are compared to each other. Moreover, sex and previous experience of the horses should be considered as influencing factors when assessing behavior during novel object tests.

## Data Availability

The raw data supporting the conclusions of this article will be made available by the authors, without undue reservation.
